# Editorials

**Published:** 1910-07

**Authors:** 


### EDITORIALS

The Business Office of the *JOURNAL-RECORD* is 1 1-2 to 5 1-2 South Broad Street.
The Editorial Office is 1014-15 Century Building.
Address all Business Communications to *Journal-Record of Medicine*, 1 1-2 to 5 1-2 South Broad Street.
Make remittances payable to *THE JOURNAL-RECORD OF MEDICINE.*
On matters pertaining to the Editorial and Original Communications, address Edgar G. Ballenger, M. D., Atlanta, Ga.
Reprints of Original Articles will be furnished at cost price. Requests for the same should always be made in THE MANUSCRIPT.
We will present, postpaid, on request, to each contributor of an original article, twenty (20) marked copies of *THE JOURNAL-RECORD OF MEDICINE* containing such article.

### DR. CLAUDE A. SMITH WINS GOLD MEDAL.

At the recent meeting of the American Medical Association, held at St. Louis, Dr. Claude A. Smith was signally honored by being awarded the gold medal for the best exhibit and paper of original research present before the committee on Scientific Exhibit and Original Research of the American Medical Association. In the exhibit Dr. Smith showed a series of large photographs of his experiment of successfully inoculating a patient by placing mud containing the hook worm larvae on the skin, thus demonstrating the mode of entrance of the hook worm into the system. Also pictures of the eruption caused on the skin by an extract of hook worm lavae. There were also interesting photographs of individuals infected with hook worms and family groups of people in the hook worm districts. He showed specimens of male and female hook worms and sand containing the lavae. There were drawings and micro-photographs of the eggs in various stages of development and the lavae. The exhibit attracted a large crowd who showed great interest in it. Among those who were especially enthusiastic in their praise of Dr. Smith's good work were Dr. Wm. T. Councilman, of Boston; Prof. Henry B. Ward, Professor of Zoology at University of Illinois; and Dr. Wm. H. Welch, President of the American Medical Association. Dr. Smith has received a number of requests from medical societies in various parts of the country asking him to come and talk on this very interesting subject of hook worm.

We congratulate Dr. Smith upon the well-deserved honor which he has received, and Atlanta is to be congratulated for having a man here whose work is recognized and honored throughout the country. —F. G. H.

---

### HYDROTHERAPY.

Among all the recent advances in the practice of medicine none is more striking than the use of water in the treatment of disease. Hydrotherapy has long been looked upon as a resource of quacks or men of one idea and has therefore struggled for the place it deserves in the field of usefulness in caring for disease. This struggle is noted when one looks for a description of its methods of application in the text-books. Almost no writer takes it up succinctly and treats it with any degree of fullness. It has remained for a few individuals to apply it scientifically and to teach its use to those who came to their clinics. These careful and thoughtful men have recently placed it beyond a peradventure as a highly beneficial agent in therapeutics and the articles written about it in various journals have demonstrated this beyond question. However, for a general practitioner there is little or no means of learning all the advances and he is handicapped when attempting to use Hydrotherapy by the necessity of working out his own experiments.

We are glad to call the attention of our readers to a series of articles which will appear from time to time in this journal written by Dr. L. Amster, of Atlanta, who, having familiarized himself thoroughly with the technique in Vienna and in other European cities and having used his methods extensively in his own practice, is able to place before the profession simple and fundamental means through which any physician may use Hydrotherapy. These articles will take up definitely the standard of the principles of Hydrotherapy and the variations either way from this standard by which a wide range of effects can be produced and the conditions of disease readily met. We hope that the use of this agent will be more generally taken up and that reports will be made as to its general application.

—R. R. D.

### CRAWFORD W. LONG THE DISCOVERER OF ETHER ANESTHESIA.

It was indeed an inspiring sight to witness the unveiling of the monument to Dr. Long, the discoverer of anesthesia, and one of Georgia's most noted citizens, at the recent meeting of the Medical Association of Georgia at the special meeting at Jefferson. The immense crowd of physicians and friends, and the near relatives of Long's, with the unstinted enthusiasm for a noble man whose discovery has probably lessened human suffering more than any other has ever done, was a sight long to remain memorable in the minds of those present. A large number of addresss were made near the site where ether was first given, in which the details of the discovery were thoroughly reviewed. There is now no further doubt as to who was the first to use ether for surgical anesthesia and Georgia is proud to claim its discoverer.

---

### DR. GEO. H. NOBLE'S NEW SANATARIUM.

Dr. Geo. H. Noble has recently completed his new sanatarium which is so elaborate and convenient in its details for a perfect technic that we think it calls for especial commendation. It is most unusual indeed to see hospital plans which have been studied out with such infinite care. Having been for years in major gynecologic surgery in his own institution, Dr. Noble seems to have had an ideal, to the attainment of which he was working and planning. This new building is the fruition of the ideas which made up his ideal hospital. At every turn from basement to top floor one is confronted with the most modern, sanitary and convenient modes of providing surgical cleanliness and comfort. The heating, the plumbing, the sterlizers, the special closets the floors, the lights, the "chutes" for soiled linen, the special sterilizers for bed pans, the shower baths for operators, the ample up-stair porches, and the splendidly arranged kitchen with special and complete diet kitchens on each floor, bear incontestable evidence, foresight and experience. In such an institution the surgeon finds it comparatively easy to carry out perfect technics and handle expeditiously the most troublesome conditions. With these facilities and Dr. Noble's well-known skill, no one will be surprised at his splendid results.

---

### DR. DUNBAR ROY HONORED BY THE AMERICAN MEDICAL ASSOCIATION.

It is with distinct pleasure we note the election of Dr. Dunbar Roy, of Atlanta, as chairman of the section of the American Medical Association on Laryngology and Otology. To be selected to this position by such a representative body of men is a mark of esteem of which no man can feel indifferent, and we heartily congratulate Dr. Roy upon this signal recognition of his ability.

